# Better post-operative outcomes at 1-year follow-up are associated with lower levels of pre-operative synovitis and higher levels of IL-6 and VEGFA in unicompartmental knee arthroplasty patients

**DOI:** 10.1007/s00167-023-07503-y

**Published:** 2023-07-14

**Authors:** Mei Lin Tay, Scott M. Bolam, A. Paul Monk, Sue R. McGlashan, Simon W. Young, Brya G. Matthews

**Affiliations:** 1 Department of Surgery, Faculty of Medical and Health Sciences (FMHS), University of Auckland, Private Bag 92-019, Auckland, 1023 New Zealand; 2 Department of Orthopaedic Surgery, North Shore Hospital, Private Bag 93-503, Auckland, 0620 New Zealand; 3 Department of Orthopaedic Surgery, Auckland City Hospital, Private Bag 92-024, Auckland, New Zealand; 4 Auckland Bioengineering Institute, University of Auckland, Private Bag 92-019, Auckland, 0620 New Zealand; 5 Department of Anatomy and Medical Imaging, University of Auckland, Private Bag 92-019, Auckland, 0620 New Zealand; 6 Department of Molecular Medicine and Pathology, University of Auckland, Private Bag 92-019, Auckland, 0620 New Zealand

**Keywords:** Unicompartmental knee arthroplasty, UKA, Medial UKA, Osteoarthritis, OA phenotype, Inflammatory marker, Synovitis, Patient selection, Biomarkers, Cytokine

## Abstract

**Purpose:**

Osteoarthritis (OA) is associated with inflammation, and residual inflammation may influence outcomes following knee arthroplasty. This may be more relevant for patients undergoing unicompartmental knee arthroplasty (UKA) due to larger remaining areas of native tissue. This study aimed to: (1) characterise inflammatory profiles for medial UKA patients and (2) investigate whether inflammation markers are associated with post-operative outcomes.

**Methods:**

This prospective, observational study has national ethics approval. Bloods, synovial fluid, tibial plateaus and synovium were collected from medial UKA patients in between 1 January 2021 and 31 December 2021. Cytokine and chemokine concentrations in serum and synovial fluid (SF) were measured with multiplexed assays. Disease severity of cartilage and synovium was assessed using validated histological scores. Post-operative outcomes were measured with Oxford Knee Score (OKS), Forgotten Joint Score (FJS-12) and pain scores.

**Results:**

The study included 35 patients. SF VEGFA was negatively correlated with pre-operative pain at rest (*r* − 0.5, *p* = 0.007), and FJS-12 at six-week (*r* 0.44, *p* = 0.02), six-month (*r* 0.61, *p* < 0.01) and one-year follow-up (*r* 0.63, *p* = 0.03). Serum and SF IL-6 were positively correlated with OKS at early follow-up (serum 6 weeks, *r* 0.39, *p* = 0.03; 6 months, *r* 0.48, *p* < 0.01; SF 6 weeks, *r* 0.35, *p* = 0.04). At six weeks, increased synovitis was negatively correlated with improvements in pain at rest (*r* − 0.41,* p* = 0.03) and with mobilisation (*r* − 0.37, *p* = 0.047).

**Conclusion:**

Lower levels of synovitis and higher levels of IL-6 and VEGFA were associated with better post-operative outcomes after UKA, which could be helpful for identifying UKA patients in clinical practice.

**Level of evidence:**

Level IV case series.

**Supplementary Information:**

The online version contains supplementary material available at 10.1007/s00167-023-07503-y.

## Introduction

Knee osteoarthritis (OA) is a leading cause of pain and disability in adults, with an estimated 240 million individuals worldwide living with symptomatic disease [1]. OA is characterised by progressive cartilage and bone degradation. Initially considered a ‘wear and tear’ disease from mechanical stresses, it is now recognised that OA also involves joint inflammation, leading to molecular, anatomic and physiologic abnormalities [14, 20].

Elevated inflammatory cytokine and chemokine levels have been associated with increased pain in OA and joint arthroplasty patients, and with arthroplasty revisions [10, 17, 31, 32]. While studies in this area have focused on total knee arthroplasty (TKA) and total hip arthroplasty, the impact of underlying biological mechanisms may be more relevant for unicompartmental knee arthroplasty (UKA) patients who have larger remaining areas of native tissue. Identifying inflammatory cytokines and chemokines that further define the OA disease state and understanding their associations with post-operative outcomes may allow for the development of biomarkers to assist with UKA patient selection and improve patient outcomes.

The aims of this study were to: (1) characterise systemic and local inflammatory profiles for patients undergoing medial UKA using molecular, anatomic and physiologic markers and (2) investigate whether these markers of inflammation are associated with post-operative outcomes in the year after surgery. The study hypotheses were that UKA patients would have some detectable levels of inflammation associated with OA pathogenesis and that patients with higher levels of pre-operative of inflammation would have poorer post-operative outcome scores.

## Methods

### Patients and ethics

This study was carried out under ethical approval and in accordance with guidelines of the Declaration of Helsinki [34]. All patients listed for primary medial UKA surgery for primary osteoarthritis between 1 January 2021 and 31 December 2021 were eligible. Patients were excluded if the surgeon decided to perform TKA or bi-UKA intraoperatively. Blood and synovial fluid (SF) samples were collected prior to surgery, whereas tibial plateau and synovium were collected intraoperatively. Patient radiographs were graded using the Kellgren–Lawrence (K–L) score [18].

The surgeries were performed by 12 consultant surgeons, and the implants used were RESTORIS^®^ MCK (*n* = 20, Stryker, Fort Lauderdale, FL, USA), Oxford Unicompartmental Knee (*n* = 7, Zimmer Biomet, Warsaw, IN, USA), Persona Partial Knee (*n* = 6, Zimmer Biomet) and Zimmer Unicompartmental Knee (*n* = 2, Smith & Nephew, Memphis, TN). All patients were offered standard of care rehabilitation classes following surgery.

### Cytokine and chemokine measurements

Bloods were collected in Vacutainer^®^ tubes with silica (BD, Franklin Lakes, NJ, USA) and processed 3–4 h post-collection. Following clotting, the samples were centrifuged at 13,000×*g* for 10 min, and supernatant frozen at − 80 °C. SF was collected in 50-mL Falcon tubes (Corning, Corning, NY, USA) and processed 2.5–4 h from collection. Samples were centrifuged at 2000×*g* for 10 min, and supernatant was frozen at − 80 °C.

Serum and SF supernatants were thawed on ice and centrifuged at 10,000×*g* for clarification. SF samples were treated with 20U/mL hyaluronidase (type IV-S, Sigma-Aldrich, St. Louis, MS, USA) for 30 min at 37 °C to reduce viscosity [9]. Protein levels were measured from serum and SF (neat) using a 14-plex assay (MILLIPLEX^®^ Human Cytokine/Chemokine/Growth Factor Panel A Immunology Multiplex Assay, Millipore, Merck, Kenilworth, NJ, USA) on a MagPix^®^ instrument (Luminex Corporation, Austin, TX, USA) according to the manufacturer’s instructions. The 14 analytes measured were interleukin (IL)-1α, IL-1β, IL-4, IL-5, IL-6, IL-8/chemokine (C-X-C motif) ligand (CXCL)8, IL-10, IL-17A, interferon (IFN)-γ, monocyte chemoattractant protein (MCP)-1/C-C motif chemokine ligand (CCL)2, macrophage inflammatory protein (MIP)-1α/CCL3, MIP-1β/CCL4, tumour necrosis factor (TNF)-α and vascular endothelial growth factor A (VEGFA). Standard curves were fitted using 5PL-parameter regression using xPonent software version 4.2 (Luminex Corporation). Coefficients of variation and spike recovery were considered acceptable according to the manufacturer’s instructions. Data are only shown for analytes that were consistently above the limit of detection.

### Histology

Tibial plateaus were washed with phosphate buffered saline (PBS), fixed in 10% neutral buffered formalin for three weeks and then demineralised at room temperature in 10% formic acid (Fisher Chemical, Thermo Fisher Scientific) for three months. The samples were resected into 1 cm by 4 cm pieces prior to paraffin embedding. Resections included three macroscopically identifiable regions: (1) macroscopically healthy cartilage, (2) transition zone, with partial cartilage thickness loss, and (3) lesion, with maximal or full cartilage thickness loss (supplementary material). Synovium samples were washed with PBS, fixed in 4% paraformaldehyde at room temperature overnight and then transferred to 70% ethanol prior to paraffin embedding. Blocks were sectioned to 5 µm using a microtome. Three sections taken 20 µm apart were placed on Epredia™ Superfrost glass slides (Thermo Fisher Scientific). Tibial plateau sections were rehydrated with xylene and ethanol, then stained with Safranin O and Fast Green. Synovium sections were rehydrated and then stained with haematoxylin and eosin. The sections were then air-dried, mounted and imaged using a slide scanner optical microscope (Vslide, MetaSystems, Altlussheim, Germany). Images were scored by two blinded observers using the Mankin histological grading system [27] and a validated synovitis scoring system [21]. The mean Mankin score for each region was calculated out of 14 points, whereas the mean synovitis score for each patient was calculated and rounded to the closest grade (maximum grade of 3). All patients had medial K–L scores of 3–4 and lateral K–L scores of 0–2, whereas patellofemoral K–L scores ranged between 1 and 3. Histological analysis indicated that Mankin scores in ‘healthy cartilage’ were 5.3 ± 2.5, transition zone 9.2 ± 2.4 and lesion 12.5 ± 2.5 (observer intraclass correlation coefficients (ICC), 0.92). All patients had Grade 1 (13 patients, 43%) or Grade 2 (17 patients, 57%) synovial scores (ICC 0.86).

### Patient-reported outcomes

The OKS measures patient pain and function on a scale of 0 (worst) to 48 (best) [29], the Forgotten Joint Score (FJS-12) measures awareness of an artificial prosthesis on a scale of 0 (worst) to 100 [5], whereas the visual analogue scale for pain (VAS-pain) measures level of pain on a scale from 0 (worst) to 100 (best) [12]. Scores were collected from UKA patients before surgery (baseline), and at six weeks, six months and one year following surgery by a research coordinator who was not involved in patient care. Post-operative ‘change’ scores compared with baseline (pre-operative) were calculated for OKS and VAS-pain.

At one-year follow-up, there was improvement in all mean change scores compared with baseline (OKS 17.5 ± 8.3, range – 11 to 30; VAS-pain at rest, 25.4 ± 29.9, range – 45 to 73; VAS-pain when mobilising, 46.2 ± 30.1, range – 35 to 89 (Supplementary material). There was also improvement for FJS-12 scores at 1-year follow-up (63.2 ± 27.8, range 16.7–100 out of 100).

### Statistical analyses

Using conservative estimates of the minimal clinically important difference (MCID) for the Oxford Knee Score (OKS) of 5 [8] and estimated population standard deviation of 7 [3], a sample size of 33 patients was required to provide enough power to detect differences between groups (power 80%, *α* = 0.05). Data were analysed using SPSS Statistics version 26 (IBM corp., Armonk, NY) and PRISM 8 (GraphPad, San Diego, CA. For categorical data, differences were determined using Fisher’s or Chi-squared tests. For continuous variables, differences were determined using *t* tests or one-way ANOVA for normally distributed variables, and Mann–Whitney or Kruskal–Wallis tests were used for nonparametric variables. Associations were assessed using Pearson’s correlation coefficient for normally distributed variables and Spearman’s correlation coefficient for nonparametric variables. *p* values below 0.05 were considered significant. For the two bilateral UKA patients, samples from the contralateral knees were treated as independent samples.

## Results

### Patients

**Fig. 1:**
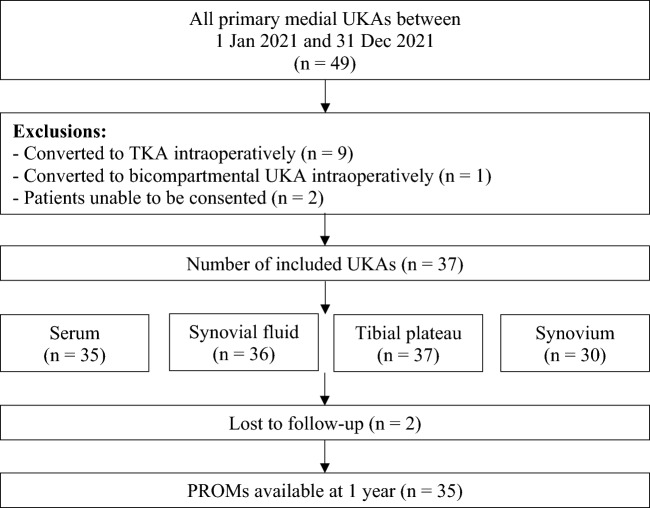
Flow chart of medial UKA cases included in the study. *PROMs* patient-reported outcome measures, *TKA* total knee arthroplasty, *UKA* unicompartmental knee arthroplasty

A total of 35 patients (37 knees) were included in this study (Fig. 1). The patients were 73% male, with a mean age of 65.7 and mean BMI of 28.9 (Table 1). None of the patients underwent any reoperations during the follow-up period.

**Table 1:** Characteristics of patients undergoing medial UKA included in this study

Total	
Knees	37
Patients	35
Gender	
Male	27 (73)
Female	10 (27)
Age at surgery (years)	
Mean ± sd	65.7 ± 9.0
Range	40–86
BMI	
Mean ± sd	28.9 ± 5.4
Range	20–48
ASA status	
1	3 (8)
2	23 (62)
3	11 (30)
Laterality	
Left	18 (49)
Right	19 (51)
Implant	
Restoris	20 (54)
OUK	9 (24)
Persona	6 (16)
ZUK	2 (5)
Fixation	
Cemented	30 (81)
Uncemented	7 (19)
Centre	
1	27 (73)
2	10 (27)

Values presented as* n* (%) or mean ± standard deviation (sd)

*ASA* American society of anesthesiologists, *BMI* body mass index, *NZ* New Zealand, *OUK* Oxford unicompartmental knee, *ZUK* Zimmer unicompartmental knee

### Pre-operative associations of molecular, anatomic and physiologic markers

**Fig. 2:**
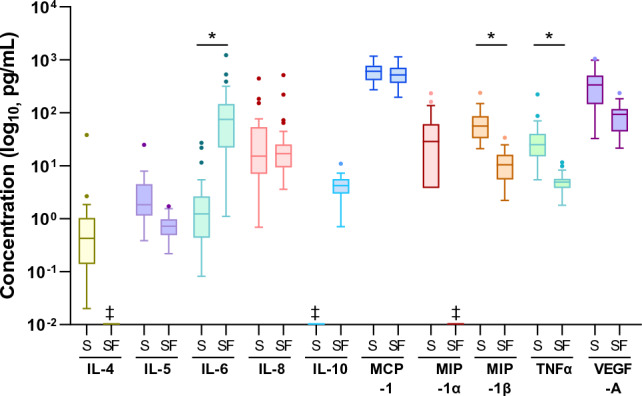
Concentrations of chemokines and cytokines in serum (S) and synovial fluid (SF) of patients undergoing primary medial UKA. *IL* interleukin, *MCP* monocyte chemoattractant protein, *MIP* macrophage inflammatory protein, *LDL* lower detection limit, *S* serum, *SF* synovial fluid, *TNF* tumour necrosis factor, *VEGF* vascular endothelial growth factor. ^‡^below detection limit, *p < 0.05, Kruskal–Wallis with Dunn’s multiple comparisons

Concentrations of selected SF, but not serum, cytokines/chemokines correlated with histological and radiographic disease measures (Fig. 2, supplementary material). Patients with a higher synovial grade, representing higher levels of synovitis, had higher SF levels of IL-8 (*r* 0.48, *p* < 0.01), IL-10 (*r* 0.41, *p* = 0.03) and MIP-1β (*r* 0.40, *p* = 0.03; Table 2).

**Table 2:** Associations between the anatomic features of OA and pre-operative inflammation cytokines/chemokines from the synovial fluid of patients undergoing UKA

SF markers	Medial K–L		Lateral K–L		PFJ K–L		Mankin ‘healthy’		Mankin ‘transition’		Mankin ‘lesion’		Synovial score	
	*r*	*P*	*r*	*P*	*r*	*p*	*r*	*p*	*r*	*p*	*r*	*p*	*R*	*p*
IL-5	0.24	n.s.	− 0.13	n.s.	− 0.20	n.s.	0.05	n.s.	0.10	n.s.	0.12	n.s.	0.34	n.s.
IL-6	0.32	n.s.	0.23	n.s.	0.10	n.s.	0.26	n.s.	**0.46**	< 0.01*	0.10	n.s.	0.01	n.s.
IL-8	**0.46**	< 0.01*	0.19	n.s.	− 0.09	n.s.	0.15	n.s.	**0.44**	0.01*	**0.39**	0.02*	**0.48**	< 0.01*
IL-10	0.12	n.s.	0.07	n.s.	0.04	n.s.	0.09	n.s.	0.19	n.s.	0.10	n.s.	**0.41**	0.03*
MCP-1	0.30	n.s.	0.10	n.s.	0.04	n.s.	− 0.03	n.s.	0.06	n.s.	0.11	n.s.	0.23	n.s.
MIP-1β	0.23	n.s.	0.02	n.s.	− 0.24	n.s.	0.12	n.s.	0.34	n.s.	**0.44**	< 0.001*	**0.40**	0.03*
TNF-α	0.23	n.s.	0.29	n.s.	0.12	n.s.	− 0.06	n.s.	0.18	n.s.	0.20	n.s.	0.24	n.s.
VEGFA	− 0.11	n.s.	− 0.10	n.s.	0.30	n.s.	− 0.13	n.s.	− 0.03	n.s.	0.05	n.s.	− 0.11	n.s.

Parameters in bold and with an asterisk are statistically significant (*p* < 0.05)

*IL* interleukin, *K–L* Kellgren–Lawrence, *MCP* macrophage colony stimulating factor, *MIP* macrophage inflammatory protein, *n.s.* not significant, *p p* value, *PFJ* patellofemoral joint,* r* Spearman’s correlation coefficient, *TNF* tumour necrosis factor, *VEGF* vascular endothelial growth factor

### Associations between pre-operative markers of inflammation and post-operative outcomes

**Fig. 3:**
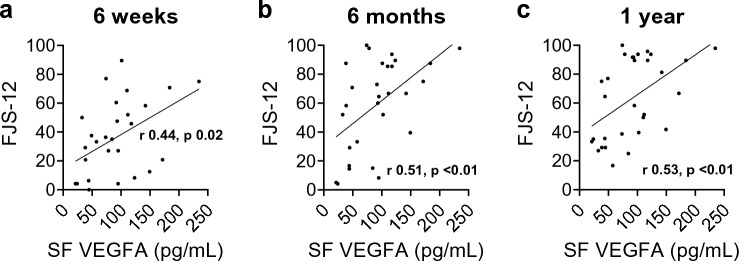
Associations between VEGFA measured in synovial fluid of patients undergoing UKA and FJS-12 score at **a** six-week, **b** six-month and **c** one-year follow-up. *FJS* forgotten joint score, *r* Spearman’s correlation coefficient, *SF* synovial fluid, *VEGF* vascular endothelial growth factor, *UKA* unicompartmental knee arthroplasty

**Fig. 4:**
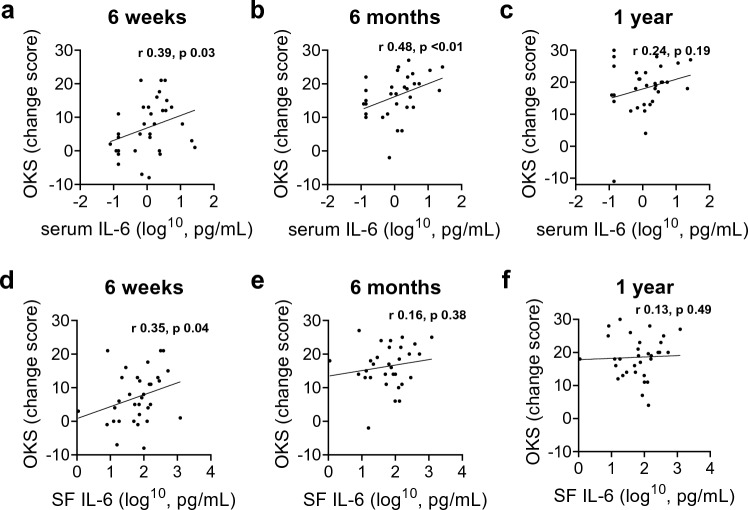
Associations between IL-6 measured in serum (**a**–**c**) and synovial fluid (**d**–**f**) of patients undergoing UKA and OKS score at (**a**), **d** six-week, **b**, **e** six-month and **c**, **f** one-year follow-up. *IL* interleukin, *OKS* Oxford knee score, *r* Spearman’s correlation coefficient, *SF* synovial fluid, *UKA* unicompartmental knee arthroplasty

Pre-operative SF VEGFA was positively correlated with FJS-12 scores at all time points following surgery (6 weeks, *r* 0.44, *p* = 0.02; 6 months, *r* 0.51, *p* < 0.01; 1 year, *r* 0.53, *p* < 0.01; Fig. 3). Pre-operative serum IL-6 was positively correlated with improvements in OKS at six weeks (*r* 0.39, *p* = 0.03) and six months (*r* 0.48, *p* < 0.01; Fig. 4A–C). Pre-operative SF IL-6 was positively correlated with improvements in OKS at six weeks (*r* 0.35, *p* = 0.04) (Fig. 4D–F). The baseline synovial score was negatively correlated with improvements in pain levels at rest (*r* − 0.41, *p* = 0.03) and with mobilisation (*r* − 0.37, *p* = 0.047) at the six-week follow-up.

## Discussion

The key finding of this study was that lower levels of pre-operative synovitis and higher levels of IL-6 and VEGFA were associated with better post-operative outcomes in UKA patients. Although OA is typically classified as a non-inflammatory disease, high prevalence of synovitis in OA patients suggests a role of synovial inflammation in pathogenesis of the disease [23, 35, 41]. In this patient cohort, more severe synovitis at surgery was associated with smaller improvements in pain scores at rest and with mobilisation at early follow-up (six weeks), but not at later follow-up. This suggests that OA patients with higher levels of pre-operative synovial inflammation may also have slower early pain recovery following UKA surgery.

Higher SF levels of VEGFA were associated with higher pre-surgical pain, but were positively correlated with FJS-12 scores at all follow-up time points, suggesting that patients with higher local levels of VEGFA before surgery achieved a joint that felt more natural. Achieving a more natural joint following knee arthroplasty is desirable, as it is correlated with patient satisfaction, and is one of the benefits of UKA compared with TKA [5, 33, 40]. VEGFA is an angiogenic factor that can promote tissue healing [2], and there is also evidence of its role in cartilage remodelling and endochondral bone formation [11]. The study findings suggest that higher pre-operative levels of VEGFA may be associated with improved remodelling and faster healing after UKA.

Pre-operative levels of local and systemic IL-6 were positively associated with better early pain and function scores, measured by OKS, after surgery. The results from this study suggest that higher levels of IL-6 prior to surgery can lead to better early post-operative outcomes. Previous studies suggest that IL-6 has a dual role in the regulation of chondrocyte function and cartilage degradation [39]. IL-6 signalling can stimulate matrix metalloprotease protein expression [22] and increase proteoglycan degeneration by chondrocytes [15], which increases cartilage degradation. In contrast, IL-6 signalling can also increase expression of the tissue inhibitor of metalloproteinases (TIMPs) [24] and stimulate proteoglycan synthesis in chondrocytes [37], which have protective effects on cartilage. Although the roles of IL-6 in joint pathology are currently not well understood, the findings from this present study suggests that the cytokine plays an important role in post-surgical cartilage homeostasis and remodelling processes.

Although higher levels of synovial inflammation were correlated with worse post-operative outcomes, as hypothesised, higher levels of the inflammatory markers IL-6 and VEGFA were associated with improved outcomes after UKA. The higher levels of these cytokines may promote post-surgical healing through improved cartilage and bone remodelling. Further research focused on VEGFA and IL-6 will help define their roles in recovery following UKA.

In this study, the inflammatory profile for patients undergoing primary UKA was characterised. IL-5, IL-6, IL-8, MCP-1, MIP-1β, TNF-α and VEGFA were detected in serum and SF of UKA patients. Only local cytokines (IL-6, IL-8, MIP-1β) showed associations with radiographic and histological anatomic measurements, suggesting that while systemic inflammation may also be involved in OA pathogenesis [7, 19], local inflammation is likely more important. While elevated circulating levels of IL-8, MCP-1, TNF-α and VEGFA [13, 19, 25, 26], and local IL-5, IL-8, IL-10, MCP-1, MIP-1, TNF-α and VEGFA [4, 19, 30, 38] have also been reported in other OA patient groups compared with non-OA controls, there are conflicting reports of IL-6 levels in terms of positive [4, 28] and negative [16, 30] associations with OA. Elevated IL-1β and IL-17 have also been reported in OA patients [7, 19]; however, these cytokines were below the detection limit of the assay in this present study, suggesting they were not elevated in this UKA patient subset. These findings were in line with the study hypothesis that UKA patients would have some detectable levels of inflammation associated with OA pathogenesis.

This study had several limitations. First, this was an exploratory study with a small sample size and there was some heterogeneity in implants used, which limited subgroup and multivariate analyses. However, the statistically significant findings provides a practical framework that can be used for future research with larger patient cohorts. Second, the outcomes of surgery were limited to one-year follow-up. However, others have reported that one-year follow-up PROMs can sufficiently capture post-operative outcomes, with minimal change in scores occurring after the first year [6]. OKS as early as six months following surgery can be used to predict subsequent revision [36]. Third, only pre-operative cytokine and chemokines were measured; therefore, longitudinal associations could not be assessed. Although this was not an aim of this study, it may be informative for future studies to investigate the longitudinal associations to better understand the role of inflammation on patient recovery. Finally, as this was the first study characterising the inflammatory profile of UKA patients, there was limited scope for comparison to other patient cohorts. Future studies could expand the study design to include healthy donors, UKA and TKA patients.

## Conclusions

Lower levels of synovitis and higher levels of IL-6 and VEGFA were associated with better post-operative outcomes after UKA, which could be helpful for identifying UKA patients in clinical practice. Future biomarker research should focus on these markers for optimisation of UKA patient selection.

## Supplementary Information

Below is the link to the electronic supplementary material.

Supplementary file1 (DOCX 4014 KB)

## Data Availability

The participants of this study did not give written consent for their data to be shared publicly, so due to the sensitive nature of the research raw data is not available. The authors confirm that pooled data supporting the findings of this study are available within the article and its supplementary materials.
