# Supplementary material for: Better post-operative outcomes at 1-year follow-up are associated with lower levels of pre-operative synovitis and higher levels of IL-6 and VEGFA in unicompartmental knee arthroplasty patients
Source: Knee Surg Sports Traumatol Arthrosc. 2023 Jul 14;31(10):4109–16. doi: 10.1007/s00167-023-07503-y (PMC10471720; doi:10.1007/s00167-023-07503-y)
Supplement: Supplementary file 1 — Supplementary file1 (DOCX 4014 KB) [file 167_2023_7503_MOESM1_ESM.docx]

**Additional File**

**Figure** Tibial plateau resection.

Red dotted lines indicate longitudinal cuts with three macroscopically identifiable regions: (1) macroscopically healthy cartilage, (2) transition zone, with partial cartilage thickness loss, and (3) lesion, with full cartilage thickness loss.


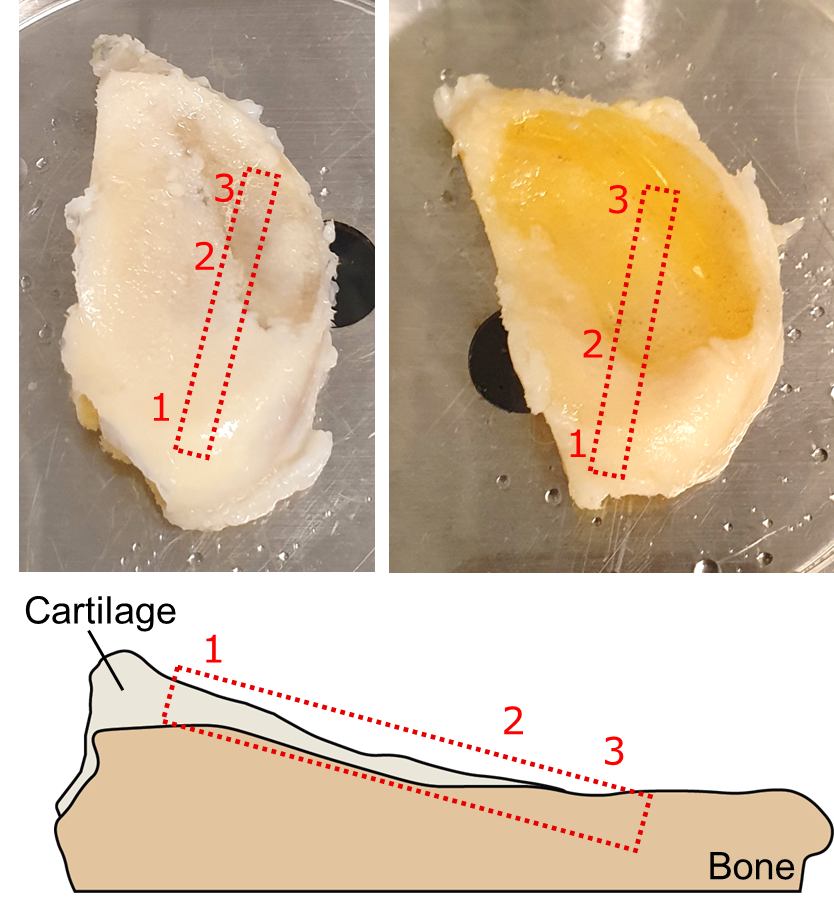


**Table** Concentrations of chemokines and cytokines in serum and synovial fluid of patients undergoing primary medial UKA

.

| Analyte | Serum (n=35) | SF (n=35) | Ratio |
| --- | --- | --- | --- |
|  | concentration (pg/mL)^a^ | concentration (pg/mL)^a^ | SF:serum |
| IL-4 | 1.75 ± 6.52 | *Below LDL* | - |
| IL-5 | 3.26 ± 4.22 | 0.70 ± 0.34 | 0.2 |
| IL-6 | 3.05 ± 5.77 | 143.20 ± 221.40 | 47* |
| IL-8/CXCL8 | 43.80 ± 80.56 | 39.42 ± 90386 | 0.9 |
| IL-10 | *Below LDL* | 4.35 ± 1.91 | - |
| MCP-1/CCL2 | 607.20 ± 234.50 | 553.70 ± 235.20 | 0.9 |
| MIP-1α/CCL3 | 42.00 ± 53.91 | *Below LDL* | - |
| MIP-1β/CCL4 | 68.43 ± 46.20 | 11.53 ± 7.17 | 0.2* |
| TNF-α | 34.68 ± 37.56 | 5.03 ± 1.92 | 0.1* |
| VEGFA | 367.80 ± 261.20 | 93.26 ± 50.56 | 0.3 |

^a^Values presented as mean ± standard deviation, *p<0.05, serum vs. SF, Kruskal-Wallis with Dunn’s multiple comparisons

*CCL,* C-C motif chemokine ligand; *CXCL,* chemokine (C-X-C motif) ligand*; IL*, interleukin; *LDL,* lower detection limit; *MCP,* monocyte chemoattractant protein; *MIP,* macrophage inflammatory protein; *SF,* synovial fluid; *TNF,* tumour necrosis factor; *VEGF,* vascular endothelial growth factor

**Figure** Representative images of Safranin O and Fast Green stained longitudinal tibial plateau sections from patients undergoing UKA at: a) macroscopically healthy, b) transition, and c) lesion regions. Histological features of OA include clefts to the transitional zone (yellow triangle), chondrocyte cloning (black arrow), tidemark crossed by blood vessels (white arrow) and severe reduction of Safranin O staining (asterisk). *OA*, osteoarthritis; *UKA*, unicompartmental knee arthroplasty

**
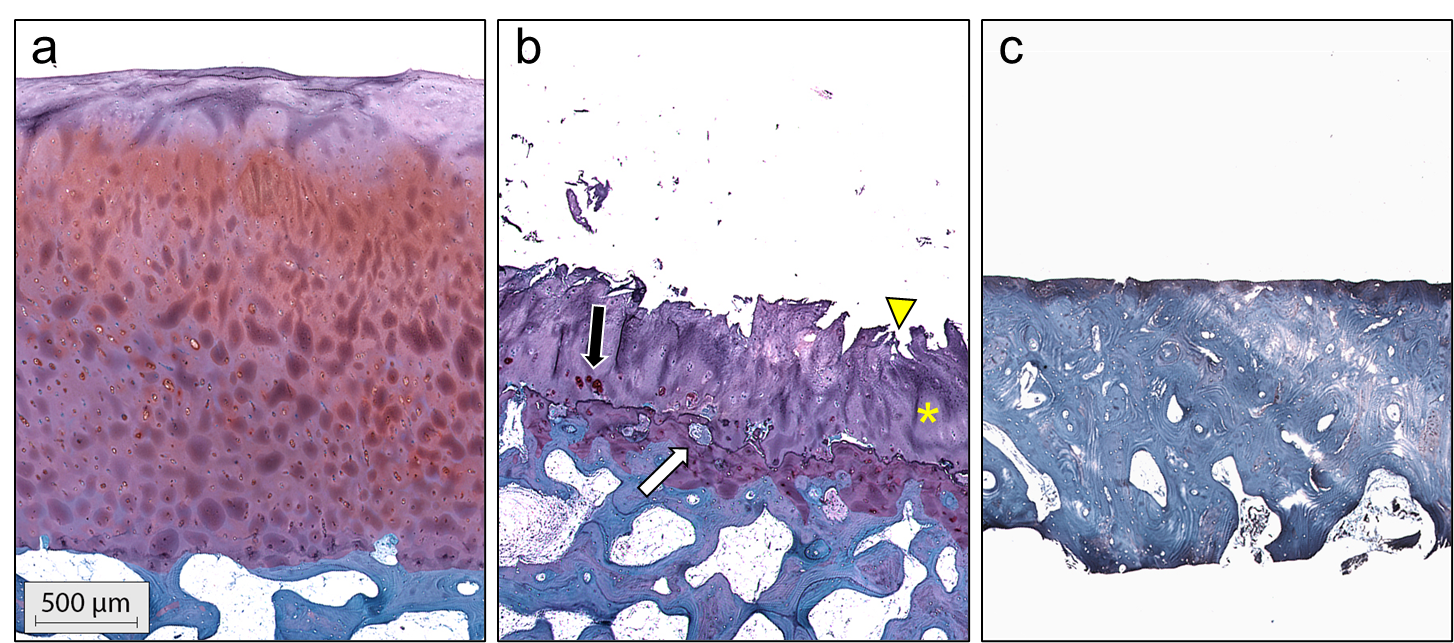
**

**Figure** Representative images of Haemotoxylin and Eosin stained sections of knee joint synovium from patients undergoing UKA, showing a) mild (Grade 1) to b) moderate (Grade 2) features of synovitis. Histological features of synovitis include moderate hyperplasia (yellow triangle) and activation of synovial stroma with vascularisation (black arrow). *UKA,* unicompartmental knee arthroplasty


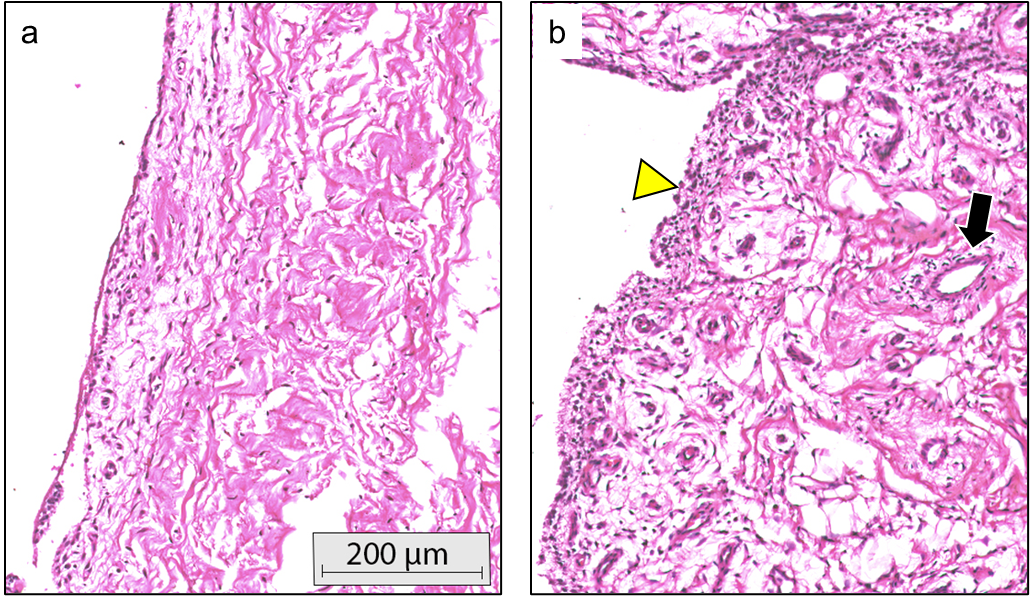


**Figure** Change in patient-reported outcome measures compared with baseline for medial UKA patients at 6-week, 6-month and 1-year follow-up for a) OKS, b) FJS-12, c) pain at rest, and d) pain with mobilisation

Values presented as mean ± standard error. Scores for OKS and pain are calculated as change from baseline (pre-operative score), with higher scores indicating better post-operative outcome in all cases. Pre-operatively, patients had mean scores of 23.1±6.3 (range 11-36 out of 48) for OKS, 43.4±23.0 (3-81 out of 100) for VAS-pain at rest, and 62.1±20.5 (23-99 out of 100) for VAS-pain when mobilising.

*FJS,* Forgotten Joint Score; *mo,* months; *OKS,* Oxford Knee Score; *w,* weeks; *y,* year.
